# The Attitudes and Professional Approaches of Dental Practitioners during the COVID-19 Outbreak in Poland: A Cross-Sectional Survey

**DOI:** 10.3390/ijerph17134703

**Published:** 2020-06-30

**Authors:** Monika Tysiąc-Miśta, Arkadiusz Dziedzic

**Affiliations:** 1 Department of Dental Prosthetics and Dental Materials, Medical University of Silesia, 40-055 Katowice, Poland; mtysiac-mista@sum.edu.pl; 2 Department of Conservative Dentistry and Endodontics, Medical University of Silesia, 40-055 Katowice, Poland

**Keywords:** COVID-19, SARS-CoV-2, dentistry, dental practitioners, attitudes, personal protective equipment, risk perception

## Abstract

The coronavirus infectious disease 2019 (COVID-19) pandemic has put enormous pressure on health care systems around the world. Dentistry has had to adjust to the new epidemic situation to not only bring relief to suffering patients but also to avoid becoming a source of SARS-CoV-2 transmission. Methods: A comprehensive, cross-sectional survey was conducted between April 6 and 16, 2020 among 875 Polish dental practitioners. The aim of the research was to assess dentists’ attitudes and professional approaches resulting from the COVID-19 pandemic. Results: 71.2% of dentists who responded to the questionnaire decided to suspend their clinical practice during that particular time. The main factors for this fact were the shortage of personal protective equipment (PPE), the respondents’ subjective perceptions of the risk of COVID-19 contraction and a general feeling of anxiety and uncertainty regarding the COVID-19 situation. The authors observed a significant decrease in the number of patients admitted weekly in April 2020 (12.06; SD, 11.55) in comparison to that in the time before the state of pandemic was declared on March 11, 2020 (49.21; SD, 24.97). Conclusions: Due to the unpreparedness of the dental sector, both in national health and private settings, most of the Polish dentists decided to voluntarily suspend their clinical practice in order to mitigate the spread of the disease. The COVID-19 outbreak has revealed numerous shortcomings in the dental care system, especially regarding the insufficient coordination of health services related to the pandemic and lack of advanced PPE. This has led to an overwhelming feeling of fear, confusion and anxiety among dental professionals in Poland and a sudden decrease in the number of performed dental procedures. Hopefully enriched with the recent experience and due to the implementation of proper strategic and long-term measures, dental practitioners will be better prepared and adapted to global health care disruptions in the future.

## 1. Introduction

From December 8, 2019, a series of pneumonia cases in Wuhan, Hubei province, China began to emerge [1]. According to data released by the World Health Organization (WHO) up to June 18, 2020, coronavirus disease 2019 (COVID-19) has affected close to 200 countries, with a total of 8,223,454 confirmed cases and 444,813 deaths worldwide [2]. On the same day, the overall number of confirmed cases in Poland reached 30,195 [3]. On 3 June 2020, the Polish Ministry of Health (PMH) announced that since the beginning of the pandemic, 1659 nurses, 660 doctors and 85 midwives had been infected with SARS-CoV-2 [4].

By the nature of their profession, dentists are exposed to pathogens located in patients’ oral cavities and respiratory tracts. Regarding the specificity of dental procedures, which involve close proximity and face-to-face contact, and the utilization of prolonged aerosol-generating procedures (AGPs), dentists’ risk of COVID-19 contraction is one of the highest among all the medical professions [5]. The potential routes for the spread of a respiratory syndrome in a dental office are direct contact with the body fluids of an infected patient, the touching of environmental surfaces and instruments contaminated with the body fluids of a COVID-positive person [6], and, potentially, contact with infectious particles that have become airborne [7]. To date, there is no scientific proof that SARS-CoV-2 is transmitted through the air, but based on the trend in the increase of the number of infections and the understanding of the basic science of viral disease, and acknowledging that SARS-CoV-1 did transmit this way, we should assume the potential airborne transmission of COVID-19 [8]. In 2003, the American Dental Association (ADA) published its first recommendations regarding dental practice during a coronavirus pandemic. They correctly predicted that patients with diagnosed SARS-CoV-1 infections needing dental treatment would be highly unlikely, because transmission occurs during the incubation period, which lasts between 2 and 10 days. SARS patients were usually extremely ill and did not undergo any elective dental procedures because of their other debilitating symptoms [9]. SARS-CoV-2 patients can transmit the disease while being asymptomatic, due to the fact that COVID-19’s incubation period ranges up to 24 days [10]. 

The first laboratory-confirmed case of COVID-19 in Poland was announced on March 4, 2020. Polish authorities decided to close schools and universities from 12 March 2020. On 20 March, 2020, a state of epidemic was announced [11]. The contemporary pandemic has necessitated a revision of the existing disease prevention protocols in dental office settings. The Polish Dental Association (PDA) played an important role in introducing new procedures during the outbreak of COVID-19 in Poland, by publishing its recommendations on how to provide dental treatment on March 19, 2020 [12]. The PMH announced its guidelines on March 25, 2020 [13]. The most considerable adjustment required was the implementation of enhanced personal protective equipment (PPE) for AGPs, such as filtering facepiece (FFP) respirators, disposable fluid-resistant gowns, airtight eye protection and full face shields [14]. This was especially challenging due to the lack of their availability for purchase and the fact that their prices have skyrocketed. This pandemic has put an enormous pressure on both private dental practitioners and the already-struggling public oral health care system. In 2018, there were 7121 dental offices and clinics that provided treatment in the public sector in Poland [15]. The overall number of performed consultations and treatment visits was estimated at 34.4 million [16]. Poland has one of the lowest funding rates for public dental services in Europe. In 2019, only 2.21% of the financial resources of National Health Fund (NHF), which constitutes approximately 425 million EUR, were designated to covering oral health care expenses [17]. Poles spend over 1.7 billion EUR on dental treatment in the private sector [18]. Despite the prompt and adequate reactions of authorities, due to the necessity of implementing new procedures, enhanced PPE shortages, and the general anxiety fueled by inconsistent information about the COVID-19 pandemic on March 16, 2020, the majority of dental offices in Poland were closed by their executives. To understand the sudden actions of Polish dentists, it is important to acknowledge the parallel epidemic situation in Italy, which was widely covered by the global media. On March 15, 2020, there were 6557 new cases reported daily. The biggest number of deaths per day was noted on 28 March, 2020 (971) [19]. Soon afterward, there were tragic reports from Spain—especially, the biggest increase in the number of newly confirmed cases per day, which occurred on April 1, 2020 (9222), and the highest death rate on April 3, 2020 (950 people) [20]. Still, it was an unprecedented situation in Polish history when the vast majority of dental practitioners voluntarily decided to suspend their clinical work in order to mitigate the spread of the disease.

Whilst dentists had to face the extraordinary epidemic situation as a result of the COVID-19 crisis, the primary aim of our research was to thoroughly assess the reasons and factors that influenced dentists’ decisions regarding their professional approaches and disengagement from clinical work during the outbreak of the pandemic in Poland. We particularly made an attempt to investigate different variables, such as a lack of access to adequate, enhanced PPE; individuals’ COVID-19 contraction risk assessments; self-reported feelings of anxiety regarding the disease; gender; the acknowledgement of national guidance on how to treat patients during the recent health care crisis; and other factors such as age, years of clinical experience, marital status, having children, place of residence, risk group for coronavirus infection due to comorbidities and, finally, dentists’ acknowledgement of the professional recommendations launched by the PDA and PMH. Our secondary aim was to assess the decrease in the number of dental patients admitted in April 2020 in comparison with that in the time before the beginning of the pandemic in Poland in March 2020.

## 2. Materials and Methods 

The cross-sectional survey was conducted between the 6th and 16th of April 2020 among Polish dental practitioners. The tool utilized for data collection was a specifically designed online Google Forms questionnaire. A representative sample group of dentists was gathered through four major Facebook groups dedicated to Polish dentists: “Dentyści”, “Dentyści Ogłaszają”, “Dentyści Przypadki, Kursy i Dyskusje” and “Lekarze Dentyści NFZ”. The Polish Dental Association and twenty-four Polish District Chambers of Physicians and Dentists were contacted via e-mail and asked to share information about the study with their members, encouraging them to participate. A total of 875 Polish dentists responded to the questionnaire. The data were collected anonymously to ensure the reliability of all of the information and compliance with EU personal data protection legislation. Ethical approval from the Bioethical Committee of the Medical University of Silesia in Katowice Poland was obtained. 

### Statistical Analysis

The quantitative statistical analysis included the chi-square test for 2 × 2 tables. In justified cases, it was supported by the determination of the odds ratio, together with a 95% confidence interval and verification using the Mantel–Haenszel test. In addition, in several cases where groups had insignificant numbers, Fisher’s exact test for 2 × 2 tables was used. Additionally, the non-parametric Mann–Whitney test was implemented, and finally, the non-parametric Kruskal–Wallis test, supplemented by post-hoc tests in the variant proposed by Conover, was utilized. The test results were considered significant when *p* < 0.05.

## 3. Results

### 3.1. General Demographic Data

A group of 875 dentists submitted completed questionnaires. According to the Supreme Medical Council of the Chamber of Physicians and Dentists (SMCCPD) on May 4, 2020, there were 37,845 professionally active dentists in Poland [21]. The age of the participants of the survey ranged from 24 to 75 years (mean of 39.1; SD, 11 years). The respondents’ demographic characteristics are presented in Table 1 [21].

### 3.2. Reasons for Continuing or Suspending Clinical Work during the COVID-19 Outbreak

Dental practitioners were asked whether they were continuing their clinical work during the COVID-19 pandemic, following the implementation of special epidemic measures in March 2020. A total of 71.2% of the respondents decided to entirely suspend their dental practice. Only 28.8% of the participants declared that they had carried on with their clinical duties. The fractional distribution of the various reasons for both choices is presented in the Table 2. More than one answer could have been chosen.

### 3.3. Factors Influencing Dentists’ Decisions and Professional Attitudes

#### 3.3.1. Access to Adequate PPE

**Figure 1:**
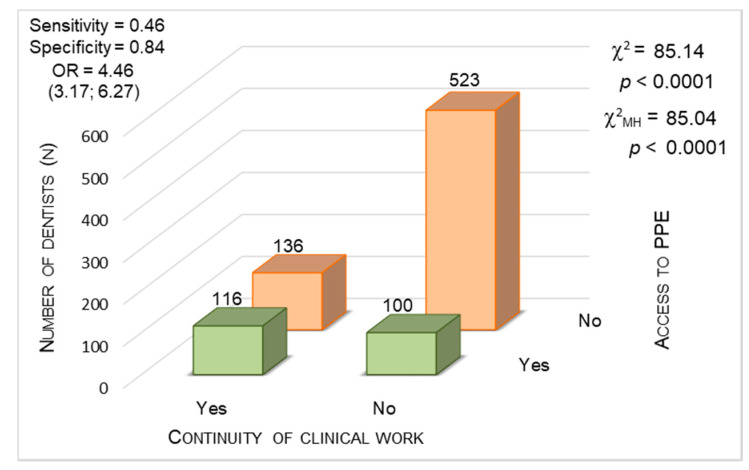
Continuity of clinical work during the COVID-19 outbreak in Poland vs. access to personal protective equipment (PPE) (*p* < 0.0001, chi-square test).

The quantitative statistical analysis included the factors that might have had influence on dentists’ decisions as to whether to work during the pandemic. A total of 75.3% of respondents said that they did not have sufficient access to PPE, while 24.7% declared the opposite. Among those who decided to work, only 46% had adequate PPE supplies; 54% stated the opposite. In the group of dentists who suspended their clinical work, 83.9% of the respondents said that they did not have sufficient access to PPE, while 16.1% were satisfied with it. The results indicated a high significance of the relationship between the decision to work during the COVID-19 pandemic and access to PPE (*p* < 0.001, chi-square test) and (*p* < 0.001, Mantel–Haenszel test). The odds ratio (OR = 4.46) indicated that dentists who continued clinical work were four and a half times more likely to have access to PPE than those who suspended their work. An average value of sensitivity (0.460) and remarkably high specificity (0.840) for the tests were obtained (Figure 1).

Dentists were also asked about the most important necessities regarding dental practice during the COVID-19 pandemic. More than one answer could have been chosen. The answers were grouped into five major categories, listed below in Table 3. 

#### 3.3.2. Subjective Risk Assessment of COVID-19 Contraction

**Figure 2:**
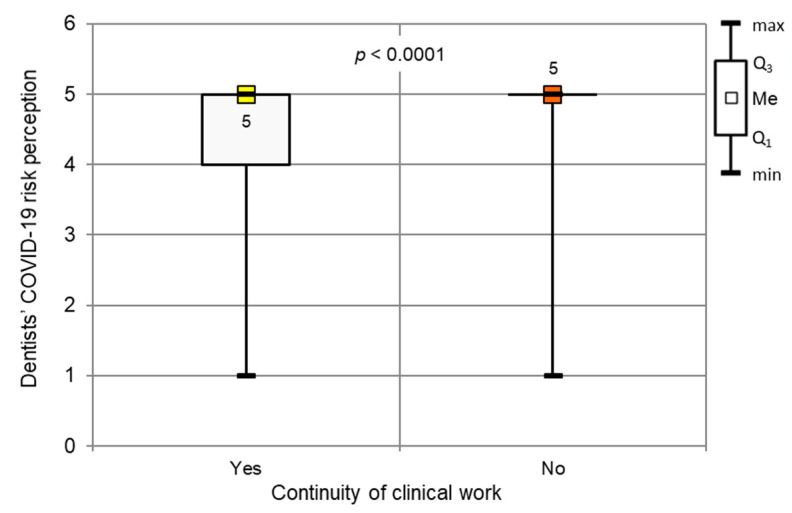
Continuity of clinical work during the COVID-19 outbreak vs. individual COVID-19 contraction risk assessment (*p* < 0.001, Mann–Whitney test).

Dentists assessed COVID-19’s occupational contraction risk as 4.77 (SD, 0.59) on a 5-point scale. Based on the Mann–Whitney test (*p* < 0.001), we found that those who did not work rated the risk significantly more highly than dentists who continued their clinical practice (Figure 2). A total of 74.8% of those who suspended their work estimated the threat as 5, whereas only 25.2% of those who continued their work rated it as high. Overall, 82.9% of all the respondents assessed the risk as 5.

#### 3.3.3. Self-Reported Anxiety Level

**Figure 3:**
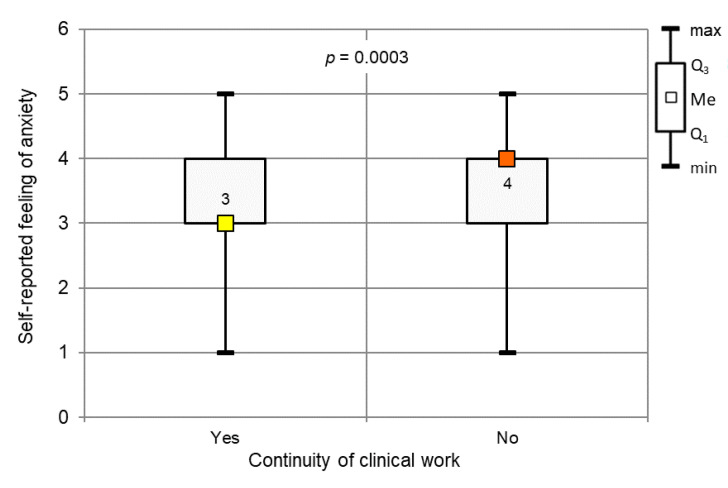
Continuity of clinical work during the COVID-19 outbreak vs. self-reported anxiety level (*p* < 0.001, Mann–Whitney test).

The respondents rated their feelings of anxiety regarding the COVID-19 pandemic as 3.61 (SD, 1.01) on a 5-point scale (Figure 3). Dentists who suspended their clinical work rated their anxiety more highly than dentists who continued their practice (*p* < 0.001, Mann–Whitney test).

#### 3.3.4. Gender

**Figure 4:**
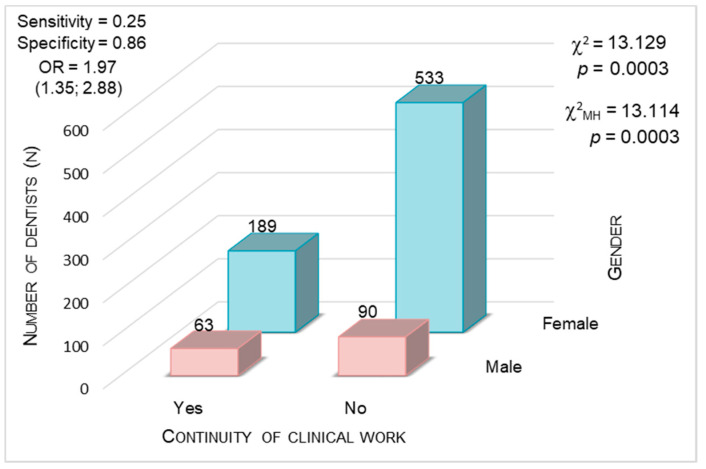
Continuity of clinical work during the COVID-19 outbreak in Poland vs. gender (*p* < 0.0001, chi-square test).

The chi-square test showed that the groups differed in terms of gender (χ^2^= 13.129; *p* < 0.001). An additional analysis was conducted by calculating the value of the odds ratio (OR) and subjecting it to verification using the Mantel–Haenszel test (χ^2^ = 13.11; *p* < 0.001). The result of this test indicates that the OR value is 1.9, which means that the chance there will be a man in the group of dentists who continued clinical work during the COVID-19 pandemic was almost twice as high as that in the group of dentists who did not continue their work. An average value of sensitivity (0.250) and remarkably high specificity (0.860) for the tests were obtained (Figure 4).

**Figure 5:**
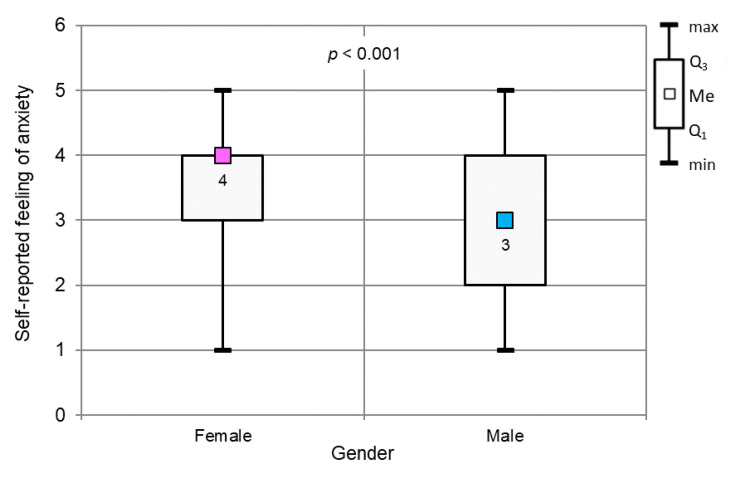
Self-reported anxiety level vs. gender (*p* < 0.001, Mann–Whitney test).

Female dentists showed a significantly higher level of self-reported anxiety (Figure 5, *p* < 0.001). 

**Figure 6:**
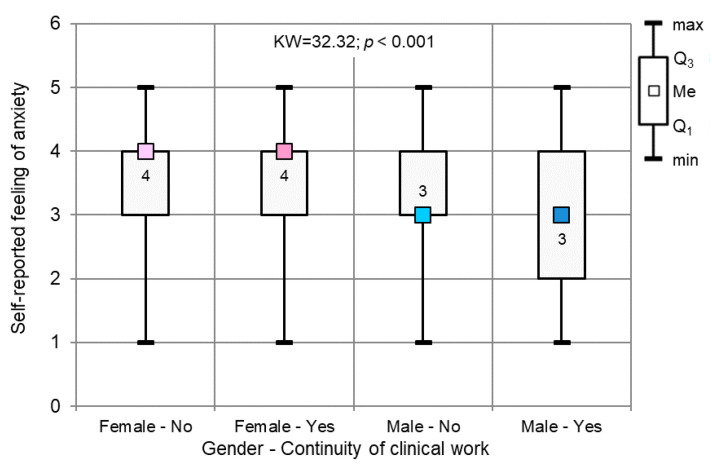
Self-reported feelings of anxiety level vs. gender: continuity of clinical work (*p* < 0.001, Kruskal–Wallis test).

The Kruskal–Wallis test confirmed (KW = 32.32; *p* < 0.001) significant differences among four groups of dentists: non-working females, working females, working males and non-working males (Figure 6). 

The Conover post-hoc test showed that the biggest difference in the self-reported feeling of anxiety occurred between the group of non-working female dentists and the group of working male dentists (*p* < 0.01) (Table 4).

#### 3.3.5. The Impact of National Guidance (PDA and PMH Guidelines)

**Figure 7:**
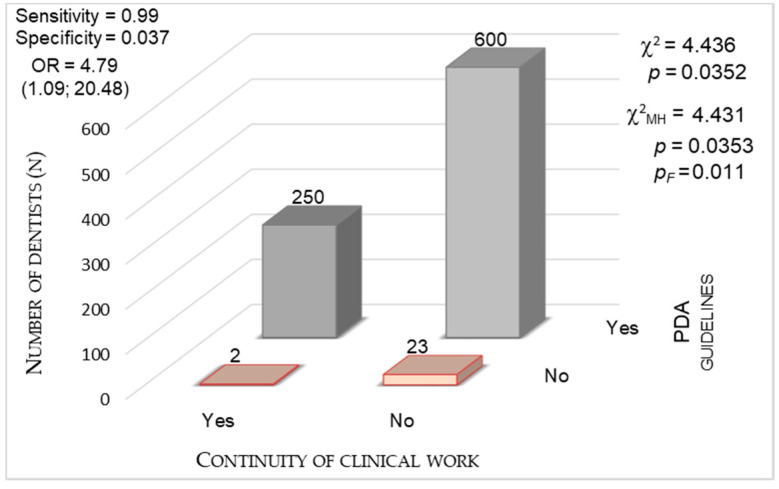
Continuity of clinical work during the COVID-19 outbreak vs. acknowledgment of Polish Dental Association (PDA) guidelines (*p* < 0.0001, chi-square test).

**Figure 8:**
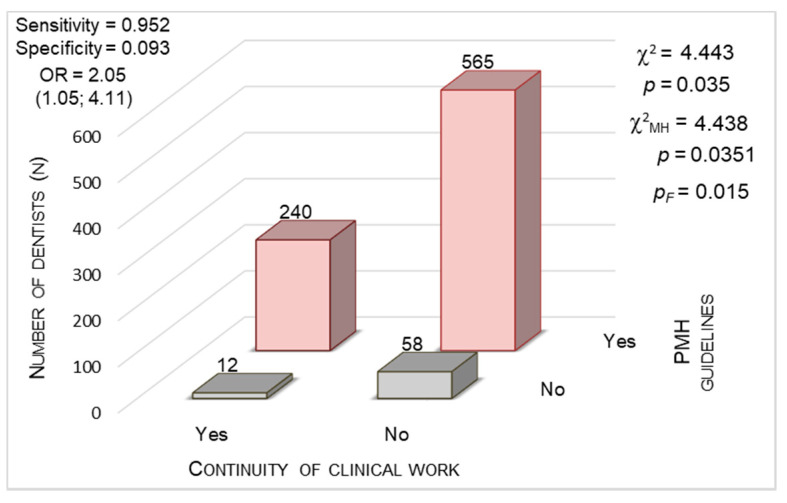
Continuity of clinical work during the COVID-19 outbreak vs. acknowledgment of Polish Ministry of Health (PMH) guidelines (*p* < 0.0001, chi-square test).

We analyzed the significance of the relationship between dentists’ decision whether to continue clinical work and acknowledgement of the PDA (Figure 7) and the PMH (Figure 8) guidelines. When assessing the PDA recommendations, the chi-square test indicated statistical significance (χ^2^ = 4.436; *p* = 0.035). The same situation occurred with that of the PMH guidelines (χ^2^ = 4.443; *p* = 0.035). The OR for the acknowledgement of the PDA recommendations reached an average of 4.8 and for that of the PMH recommendations, 2.05. It means that dentists who continued to practice were almost five times more often acquainted with the PDA guidelines and two times more often acquainted with the PMH guidelines compared to dentists who suspended their work. We noticed exceedingly high sensitivity (0.992) in detecting people who acknowledged the PDA recommendations in the group of professionally active dentists (specificity, 0.037). The values of sensitivity and specificity calculated for the acknowledgement of the PMH recommendations in the subgroups of dentists who worked clinically and those who did not were 0.952 and 0.093, respectively.

We asked our respondents how they rated the assistance of the PDA and PMH recommendations on a scale of 1 to 5. The PDA guidelines were rated as 3.33 (SD, 1.00), and the PMH guidelines, as 3.0 (SD 1.00). Dentists graded the work of the Polish Chamber of Physicians and Dentists as 2.29 (SD, 1.11) on a 5-point scale.

### 3.4. The Decrease in the Number of Admitted Patients 

**Figure 9:**
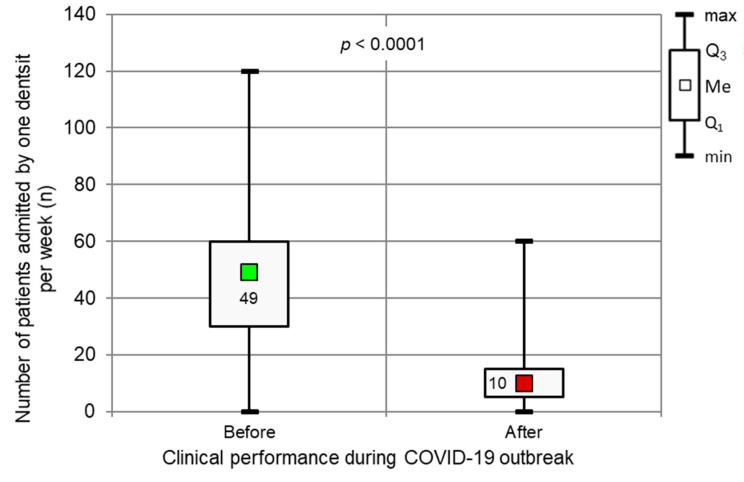
Number of patients admitted by one dentist per week, and clinical performance before and during a peak of the COVID-19 outbreak (*p* < 0.0001, Wilcoxon test).

A significant decrease in the number of patients admitted weekly by Polish dentists before and during the COVID-19 pandemic (Figure 9) was observed (*p* < 0.0001, Wilcoxon tests). We defined the period before the pandemic as the time before March 11, 2020, the day when the Director-General of the WHO officially declared the present outbreak of coronavirus disease (COVID-19) a pandemic. The period during the pandemic refers to the time frame of the conducted survey; that is, the time between April 6 and 16, 2020. The number of patients decreased from 49.21 (SD, 24.97) to 12.06 (SD, 11.55). These calculations only considered the number of patients treated by dentists who continued their clinical practice during the outbreak. In the entire group of examined dentists, the number of patients dropped from 47.13 (SD, 24.93) to 3.60 (SD, 8.31).

### 3.5. Other Investigated Factors

We also investigated factors such as age, years of clinical practice (Table 5), marital status, having children, place of residence, belonging to the risk group for coronavirus infection due to comorbidities, and dentists’ opinions on the lasting impact of COVID-19 on dental procedures (Table 6) in relation to the decision to continue dental practice or not. No statistical significance was observed.

## 4. Discussion

Our research provided an insight into reasons and factors that influenced the attitudes of Polish dentists during the COVID-19 pandemic in Poland. In our sample, women were predominant due to the fact that the number of female dentists in Poland (77%) is higher than the number of male dentists (23%) [21]. The age and place of residence distributions are slightly less representative, evidencing a sort of selection bias, probably due to the social network dissemination of the questionnaire.

On April 6, 2020, when we started to conduct the survey, there were 4201 people who had tested positive for COVID-19 in Poland; 99 of them were novel cases. Up to that day, 98 Polish citizens had died due to coronavirus infection, and 162 had recovered [22]. At the end of survey on April 16, 2020, there were 7771 confirmed cases; 189 of them were novel. Two hundred and ninety-two Poles had died and 774 had recovered up to that day. The number of cases had not reached its peak [23]. In this period, 71.2% of the respondents decided not to practice dentistry. In comparison to in other European countries, this situation was exceptional, because everywhere else, if any restrictions were imposed on the oral health care sector, they were implemented and executed by the authorities. Our findings are similar to the results of a study conducted by Ahmed and Jouhar et al. in a group of 650 dentists from 30 countries (only one from Poland), which found that 66% of respondents decided to suspend their dental practices until the number of COVID-19 cases started to decline [24].

Among the dentists who continued their clinical work during the pandemic, the main reason for their decision was the altruistic need to provide emergency and urgent dental procedures. This essential duty of medical/dental care is a fundamental principle of the dental profession. In a study conducted among 711 first year dental students from 14 countries, 36.3% declared that they decided to become a dentist to help poor and underprivileged people to improve their oral health [25]. On the contrary, the two main reasons for dentists to discontinue their clinical activities during the COVID-19 pandemic were fear for their own wellbeing and, equally, the wellbeing of their close relatives/families. Studies on earlier outbreaks of coronavirus infectious diseases such as SARS [26] and MERS [27] revealed many factors leading to psychological distress, including the fear of becoming infected while treating a patient or passing the infection on to family. In a previously mentioned study by Ahmed and Jouhar et al., 92% of dentists declared that they were afraid of carrying the COVID-19 infection from their dental practice to their families [24]. Additionally, in the study by Duruck at el., facing COVID-19 contraction threat, 90% of dentists were concerned about their families and about themselves [10]. According to the research conducted by Maunder et al., many hospital staff members during the SARS pandemic expressed conflict between their roles as health care providers and parents, feeling, on the one hand, a duty of care and, on the other hand, fear and guilt about potentially exposing their families to infection [28]. The second reason for dentists’ decisions to suspend their clinical work was the fact that many of the respondents thought that the dental surgeries were not adequately equipped, and they believed that during a pandemic, there should be special emergency dental clinics assigned by the PMH. Unfortunately, such clinics were not designated by the Polish authorities. Instead, the PDA launched a campaign—“I do not panic. I treat responsibly”—to reassure dentists that with the implementation of enhanced infection control protocols, dental treatment could be resumed [29]. The PDA also published a list of dental offices that volunteered to help patients with dental emergencies during the COVID-19 pandemic [30]. 

The subject of PPE is discussed in almost every piece of survey-based research regarding dentists during the COVID-19 pandemic. The necessity of having substantial knowledge and awareness regarding enhanced PPE utilization is emphasized. In the study conducted by Ahmed and Jouhar et al., 90% of respondents reported not wearing an N-95 mask while treating a patient. The research by Duruk et al. [10] also showed that only 12% wore an N-95 mask. Cagetti et al. reported that 55% of respondents used an FPP2 or FPP3 mask. Based on this research, we do not know if it is a result of shortages in PPE supplies or a lack of willingness to implement adequate procedures [24]. In more recent studies from Italy, it has been revealed that dentists’ attitudes regarding PPE could be improved [31]. Our research emphasizes the fact that during the time between April 6 and 16, 2020, access to PPE in Poland was extremely limited. All the PPE resources were targeted to hospitals. The authorities did not take the oral health care sector, either public or private, into consideration. According to our research, access to PPE was a particularly important decisive factor for Polish dentists as to whether to continue or suspend their clinical practice during the pandemic. In March 2020, the World Health Organization (WHO) released a press report highlighting the severe shortage of personal protective equipment (PPE) affecting health care workers worldwide during the COVID-19 pandemic [32]. There was a myriad of reports about the lack of personal protective equipment (PPE) all over the world [33,34,35], The Royal College of Surgeons of England conducted a survey on PPE between April 6 and 9, 2020, which revealed that more than half (57%) of doctors had described shortages of PPE in the past 30 days Discussions around PPE were increasingly politicized and sensitive [36], causing overwhelming anxiety both in health care professionals and patients [37]. Due to the shortages, research on refreshing face masks for extended wear and reusing them after a cleaning process emerged [38]. 

The COVID-19 outbreak had a large impact on health care providers all over the world. Until May 12, 2020, the official number of infected health workers in Italy amounted to 21,981. The number of deceased physicians reached 160, of whom 16 were dentists [39]. This reinforces the concept that close contact with positive patients, whether symptomatic or not, exposes health care workers to a higher risk of infection. SARS-CoV-2 has been demonstrated to remain aerosolized for 3 h after contamination and on plastics and stainless steel for up to 72 h. It has a half-life in aerosols that is relatively long and lasts approximately 1.1 to 1.2 hours [40]. In research by De Stefani et al., Italian dentists evaluated COVID-19 danger as 8/10 and their worries about being at risk of contagion at work as 7.3/10 [41]. In another study from Italy, 65% of responders evaluated the dentists’ infection risk as very likely [39]. These results are coherent with our findings. Most Jordanian dentists participating in a survey on COVID-19 perceived the risk as moderate, and almost one-third believed that it was not a serious public health issue; however, we have to be aware that there were no “local” cases in Jordan at the time of this data collection [42].

Anxiety, insomnia, depression, obsessive-compulsive symptoms and somatization are all well-known psychological hazards for health care workers during a pandemic [43]. Duruk et al. [10] noticed that 95% of female and 88% of male dentists were concerned about being infected with COVID-19 due to high occupational risk. According to our study, dentists who stayed at home during the outbreak also had a significantly higher level of self-reported anxiety. Our findings are consistent with a study, which showed that front-line nurses had significantly lower vicarious traumatization scores than non-front-line nurses [44]. We believe that dentists who decided to work showed better coping mechanisms, which helped them to overcome their anxieties and to provide oral health care. We also found that men were two times more likely to work during the pandemic than their female counterparts. This may be due to the fact that some women declared in the questionnaire that they were pregnant or that they had to stay at home with their children, because kindergartens and schools in Poland were closed because of the pandemic. However, we also noticed that women were more likely to suspend their clinical practice due to a self-reported feeling of anxiety. The highest level of anxiety was observed in the group of non-working women, and the lowest, in the group of working men. In the research by Choy at el., female dentists had higher mean scores for patient-related, job-related, staff-related and technical-related stressors than male dentists in everyday dental practice [45]. On the contrary, in the research by Shacham et al. [3] on factors related to the psychological distress caused by the COVID-19 pandemic among Israeli dentists and dental hygienists, gender did not have a significant impact.

In order for dentistry to do its part in mitigating the spread of COVID-19, new protocols for admitting dental patients were introduced. Our research shows a great need for PDA and PMH guidance and the notable impact of these authorities on the making of an informed decision on whether to provide dental treatment or not. The need for leadership and the feeling that one is not alone in a health care crisis of this magnitude was also emphasized by Mauder [28].

In our study, we observed a vast decrease in the number of treated patients. This is consistent with an analysis conducted in China in the period February 1–10, 2020, when the number of dental patients declined by 38%. The conclusions of that finding were that the COVID-19 situation significantly influenced people’s dental care-seeking behavior and that they were not willing to go to dental institutions for non-urgent work. Another important conclusion was that people’s need for dental services might grow explosively when the threat of COVID-19 is over [46]. This situation was the consequence of the PDA and PMH recommendations, which suspended elective dental procedures and encouraged teleconsultations and e-prescriptions, in order to minimize the number of dental patients reporting to outpatient clinics to seek treatment. The second factor was the implementation of new, necessary infection control procedures. According to new guidelines, one patient per hour should be appointed. Patient flux should be organized in such a way that only one patient in the waiting room is present [47]. Dental practices established pre-check triages to measure and record the temperature of every staff member and patient with a contact-free forehead thermometer as a routine procedure. Dental staff were required to ask patients questions about their health status regarding possible COVID-19 symptoms, and patients’ contacts needed to be provided with medical masks and hand disinfection agents once they entered the dental office [48]. If aerosol-generating procedures were impossible to avoid, dental dams, high-volume suction, and swabbing or disinfection of the teeth prior to the commencement of tooth preparation should have also been included [49]. After the procedure, all the disposable protections had to be removed, and high-level disinfection of the whole operating room with sodium hypochlorite 0.1% or 70% isopropyl alcohol performed. After each patient, an air change of at least five minutes was advised [50]. The first report on how to organize dental procedures under the premise of adequate protection measures during the COVID-19 pandemic came from the School and Hospital of Stomatology of Wuhan University, where the number of admitted patients was also reduced [6].

The COVID-19 pandemic has introduced several new problems regarding oral health care in Poland. Due to shortages in the access to PPE, their prices have rapidly increased. In May 2020, the owners of public dental offices signed a petition to the NHF with a request to revise the evaluation of dental procedures [51]. On June 2, 2020, the NFH published a draft of a new decree regarding dentistry funding. Though needs such as the increase in the funding of endodontic procedures have been recognized, the proposal was negatively assessed by the Supreme Medical Council (SMC) on June 8, 2020. According to the SMC, the proposed changes will only slightly increase the valuation of the scheduled visits [52]. On the other hand, the inevitable increase in the prices of dental treatment in the private sector has encountered many unfavorable opinions from the news media and patients in Poland [53]. It is predicted that the coronavirus pandemic will also have a negative financial impact on the dental sector as a whole, and many practitioners might not be able to restart their practice because of the new disease prevention protocols, which require investment. This will further reduce access to primary and specialist dental care [54]. On April 22, 2020, an Association of Polish Dental Employers was established, its main goals being to represent the social and economic interests of union members and initiate activities aimed at increasing the competitiveness of union members and the quality of dental services [55]. The authors suggest that a fund, which would provide financial support to its members in moments of crisis like this, should be established. Shanafelt et al. identified that health care professionals tended to have five types of requests to their organization during the COVID-19 pandemic: ‘*hear me, protect me, prepare me, support me and care for me*’. It is critical that leaders understand the sources of distress, assure health care professionals that their concerns are recognized, and work to develop approaches that will help to minimize these concerns to the extent that they are able [56]. Only by re-organizing health care systems in this manner, based on empathy and the understanding of employees’ needs, would we be able to continue to have devoted and caring medical personnel. Despite these difficulties, in June 2020, the majority of dentists in Poland returned to work, implementing additional, strict infection control protocols.

Finally, as Rosenberg argued, epidemics put pressure on the societies they strike, and as a result, they provide a sampling device for social analysis. They clearly demonstrate what really matters to a population and what they truly value. The history of epidemics offers considerable advice but only if people know the history and respond with wisdom [57]. Hopefully, our research will add insight into how to reorganize dental care when future pandemics emerge. 

It is important to stress the limitations related to sampling error in this research, including the relatively moderate sample group. This could have been caused by the short period of data collection, leading to mainly dentists who were active on social media during the short period of data collection participating in the study.

## 5. Conclusions

Due to the lack of preparedness of the dentistry sector, both public and private, a substantial majority of Polish dentists decided to voluntarily suspend their clinical practice. The COVID-19 outbreak has revealed numerous shortcomings in the dental care system, especially regarding the insufficient coordination of services related to the pandemic and general deficit of advanced PPE. The direct result of the overwhelming fear, confusion and anxiety among dental staff, which was amplified by the high perception of COVID-19 contraction risk, was a significant reduction in dental clinical practice in Poland. A sudden decrease in the number of performed dental procedures and implementation of new infection control protocols has caused financial problems for many dental practices. It is expected that dentists, enriched with the experience acquired during the recent outbreak, will be able to efficiently redefine their scope of practice and adjust to the new circumstances. 

## Figures and Tables

**Table 1:** Demographic characteristics of the group of surveyed Polish dentists (n = 875).

Demographics	Variable	Dentists, n (%)
Gender	Female Male	722 (82.5%) 153 (17.5%)
Age	24–30 31–40 41–55 ≥56	255 (29.1%) 293 (33.5%) 223 (25.5%) 104 (11.9%)
Years of clinical experience	≤10 11–20 21–30 ≥31	419 (47.9%) 209 (23.9%) 169 (19.3%) 78 (8.9%)
Marital Status	Single Married Widowed Divorced	216 (24.7%) 558 (63.8%) 39 (4.5%) 62 (7.1%)
Number of children	0 1 2 ≥3	302 (34.5%) 239 (27.3%) 271 (31.0%) 63 (7.2%)
Place of residence	Urban areas Rural areas	623 (71.2%) 252 (28.8%)
Belonging to the risk group for coronavirus infection due to comorbidities (diabetes, cardiovascular diseases, respiratory diseases, autoimmune diseases, oncological diseases)	Yes No	252 (28.8%) 623 (71.2%)

**Table 2:** Reasons for continuing or suspending clinical practice during the COVID-19 pandemic in Poland.

The Reasons Why Dentists Decided to Continue Their Clinical Work During The COVID-19 Pandemic	Number Of Dentists Who Chose The Answer (N)/(%) From The Group of 252 Dentists Who Continued Clinical Practice
I do not want to leave my regular patients without help.	130 (51.6%)
I do not want to leave patients suffering from pain unattended.	151 (59.9%)
My employer instructed me to continue my clinical practice.	39 (15.5%)
I work in a sufficiently equipped dental office.	82 (30.1%)
My financial situation forces me to continue my clinical practice irrespective of the COVID-19 pandemic.	78 (31 %)
Other.	23 (9.1%)
**The Reasons Why Dentists Decided Not To Continue Their Clinical Work During the COVID-19 Pandemic**	**Number Of Dentists Who Chose the Answer (n)/% From of the Group of 623 Dentists Who Did Not Continue Clinical Practice**
I fear for my health and life.	319 (51.2%)
I fear for the health and life of the members of my family.	359 (57.6%)
I work in an insufficiently equipped dental office.	395 (63.4%)
I believe that during the COVID-19 pandemic, patients should only be admitted to dental clinics designated by the Polish Ministry of Health.	333 (53.5%)
My financial situation allows me to suspend my clinical work until the number of COVID-19 cases starts to decline.	145 (23.3%)
Patients cancelled their appointments because they are afraid of contracting COVID-19 in the dental office.	230 (36.9%)
The dental office where I am employed is closed.	219 (35.5%)
Other.	59 (9.5%)

**Table 3:** Main demands in dental practices during the peak of the COVID-19 pandemic.

Crucial Needs in Dental Practices During the COVID-19 Outbreak	Number of Dentists Who Chose The Answer (n)/% from the Group of 569 Dentists Who Answered the Question
Personal protective equipment: filtering face piece (FPP3, FPP2) respirators, fluid-resistant surgical masks, airtight googles, full face shields, scrub caps, fluid-resistant gowns, fluid-resistant aprons, shoe covers	563 (98.9%)
Disinfectants (skin, surfaces)	92 (16.16%)
Equipment (UV lamps, vaporized hydrogen peroxide fumigation systems, non-thermal plasm disinfection devices, ozone generators)	72 (12.65%)
Adapted dental offices (separate rooms for doffing and donning of PPE, efficient ventilation systems)	63 (11.07%)
Procedures (no update on disinfection and work safety protocols, lack of goodwill from management to adjust the dental office to the new procedures)	53 (9.31%)

**Table 4:** Self-reported feeling of anxiety level vs. gender: continuity of clinical work. Conover post-hoc, significant results in bold.

Conover Post-Hoc Test						
Compared subgroups	Female–No	Female–No	Female–No	Female–Yes	Female–Yes	Male–No
	Female–Yes	Male–No	Male–Yes	Male–No	Male–Yes	Male–Yes
*p*	0.0723	0.0361	0.0064	0.1709	0.0165	0.0460

**Table 5:** Continuity of clinical work during the COVID-19 outbreak in Poland vs. age and years of clinical experience. Non-parametric statistical analysis, Mann–Whitney test (*p* = 0.283; *p* = 0.324).

Variable	Statistical characteristic	Continuity of Clinical Work During the COVID-19 Outbreak in Poland		Mann–Whitney Test
		Yes	No	
Age	Mean	39.5	39	*p* = 0.283
	SD	10.9	11.1	
	Min	24	24	
	Q1	29	30	
	Median	37	36	
	Q3	48.3	47	
	Max	72	75	
Years of clinical experience	Mean	14.4	14	*p* = 0.324
	SD	11.1	11.1	
	Min	0	0	
	Q1	4	5	
	Median	12	11	
	Q3	24	23	
	Max	52	50	

**Table 6:** Continuity of clinical work during the COVID-19 outbreak in Poland vs. marital status, having children, place of residence and belonging to the risk group for coronavirus infection due to comorbidities. Non-parametric statistical analysis, chi-square test (*p* = 0.852; *p* = 0.585; *p* = 0.594; *p* = 0.148; *p* = 0.635).

Variable	Category	Continuity of Clinical Work During the COVID-19 Outbreak in Poland		Chi-Square Test
		Yes	No	
Marital status	Married	159 93	399 224	Χ^2^ = 0.035 *p* = 0.852
	Single/widowed/ divorced			
Having children	Yes	169	404	Χ^2^ = 0.298 *p* = 0.585
	No	83	219	
Place of residence	Urban areas	213	537	Χ ^2^ = 0.285 *p* = 0.594
	Rural areas	39	86	
Belonging to the risk group for coronavirus infection due to comorbidities	Yes No	55 197	167 456	Χ^2^ = 2.095 *p* = 0.148
